# Moral injury in animal care workers: prevalence, pathways, and phenomenology in a cross-sector sample

**DOI:** 10.3389/fpsyt.2026.1820899

**Published:** 2026-06-17

**Authors:** Jamie McNally

**Affiliations:** Fortifyu Consulting, Livonia, MI, United States

**Keywords:** animal care workers, animal welfare, caseness, moral distress, moral injury, occupational mental health, shelter workers

## Abstract

**Introduction:**

Moral injury, the psychological harm resulting from events that violate one’s deeply held moral beliefs, has been extensively studied in military and healthcare populations but remains largely unexamined among animal care workers.

**Methods:**

This cross-sectional survey of 291 animal care workers across six sectors (rescue, shelter, animal control, veterinary medicine, volunteer/foster, and other) provides the first empirical prevalence estimate of moral injury in this cross-sector population using a validated syndrome measure.

**Results:**

Findings from the Moral Injury Outcome Scale (MIOS) indicate that 83.5% of participants endorsed at least one morally injurious experience pathway; most common was witnessing (63.9%), followed by being affected by others’ transgressions (38.5%) and direct participation (29.2%). Applying published caseness thresholds, 18.9% of the potentially morally injurious event (pMIE) endorsers met criteria for moral injury as a clinical syndrome and an additional 21.4% for moral distress (subclinical), with blended shame and trust violation presentations characterizing the majority (53.1%) of clinical cases. Patient Health Questionnaire-9 scores exceeded the clinical threshold for moderate depression (M = 10.96, SD = 6.27). One-way analysis of variance revealed significant sector differences in functional impairment, trust violations, and depression, with shelter workers reporting the highest levels across nearly all measures. The MIOS Shame subscale was more strongly associated with both depression (r = .646) and functional impairment (r = .343) than the Trust Violations subscale, which showed no significant relationship with functional impairment (r = .045, p = .456). Endorsement of the direct participation pathway was the only exposure pathway significantly associated with caseness classification, with 31.8% of direct participation endorsers meeting criteria for moral injury compared to 12.0% of non-endorsers (p < .001). A departure from standard MIOS administration presenting impact items to all participants revealed that a small number of pMIE non-endorsers met caseness thresholds despite actively denying exposure, raising questions about the comprehensiveness of current pathway categories.

**Discussion:**

These findings identify moral injury as a prevalent and clinically significant occupational experience among animal care workers, with distinct shame-based and trust violation-based mechanisms in predicting functional decline and clinical severity, respectively. Implications for targeted intervention and organizational reform are discussed.

## Introduction

Animal care workers experience significant occupational distress, and the field’s understanding of these challenges has been shaped predominantly by constructs of burnout (1, 2), compassion fatigue (3, 4), and secondary traumatic stress (5). While attention to these constructs has meaningfully advanced awareness of worker well-being, inconsistent terminology and persistent conceptual confusion among these constructs continues to impede both research and targeted intervention (6, 7), and a growing body of evidence suggests that these frameworks may be insufficient to capture the full phenomenology of occupational harm in this population (8–10). The construct of moral injury offers a framework for understanding ethical and systemic dimensions of professional suffering that these existing constructs do not address.

Moral injury refers to the lasting psychological, behavioral, social, and spiritual harm resulting from exposure to events that transgress one’s deeply held moral beliefs and expectations (11). While many traumatic events can wound an individual physically and psychologically (12), moral injury draws attention to a distinct mechanism of harm that is rooted not in threats to one’s life but in threats to one’s values (13). A potentially morally injurious event (pMIE) is an event that has the capacity to violate an individual’s moral code and deeply held concepts of right and wrong, including acts of perpetration or omission by the self or others (11). Moral injury is conceptualized as occurring through three pathways—transgression-self, transgression-other, and betrayal—each of which carries distinct phenomenological implications (11, 14, 15) and differential consequences for moral emotions, relational behavior, and prosocial functioning (16). It has been emphasized that moral injury is best understood as a dimensional construct with clinical significance ranging from subclinical moral distress to a fully expressed clinical syndrome, an approach that has now been operationalized through validated caseness thresholds for the Moral Injury Outcomes Scale (MIOS) in military populations (17).

While moral injury was originally conceptualized in military populations (11, 18), research has increasingly recognized its relevance across civilian occupational contexts where workers routinely encounter ethically complex, high-stakes situations (19). This expansion has been most pronounced in healthcare settings, where the moral dimensions of occupational harm gained significant empirical attention during and after COVID-19 (20) and where scholars have argued that moral injury more accurately captures clinician distress than the burnout framework (21). First responders and public safety personnel represent another emerging area of focus (22), as institutional structures and operational demands in these fields regularly expose workers to potential moral harm (13, 23).

The recognition of moral injury has also recently gained broader institutional momentum. In September 2025, the American Psychiatric Association expanded Z code Z65.8 in the DSM-5-TR to include “Moral, Religious, or Spiritual Problem,” formally acknowledging that experiences which “disrupt one’s understanding of right and wrong, or one’s sense of the goodness of oneself, others, or institutions, may warrant clinical attention (24).” While moral injury remains classified as a condition that may be a focus of clinical attention rather than a formal diagnostic category, this recognition represents a significant step toward integrating moral dimensions of suffering into psychiatric and clinical practice.

As moral injury gains clinical and institutional recognition, empirical data are needed to determine its relevance across occupational populations. Moral injury prevalence data have recently become available for military and healthcare populations; Maguen and colleagues (25) reported prevalence rates across combat veterans, healthcare workers, and first responders, and Litz and colleagues (17) established caseness thresholds in a nationally representative veteran sample using the MIOS. However, no study has applied a validated moral injury syndrome measure with clinical caseness thresholds to an animal care population. While preliminary research has examined moral injury in shelter workers using adapted versions of the Moral Injury Events Scale (8), this measure assesses exposure to morally injurious events rather than the clinical syndrome of moral injury, and these studies have been limited to single sectors. The absence of syndrome-level prevalence data grounded in a validated outcome measure with clinical thresholds leaves the field without empirically grounded estimates of moral injury severity, subvariant presentation, or cross-sector variation, relying instead on proxy indicators of moral harm, such as burnout and compassion fatigue scores, which do not capture the moral-evaluative and relational dimensions central to moral injury.

Furthermore, what empirical attention has been directed toward psychological distress in animal care has been concentrated in a narrow range of sectors, leaving the broader landscape of animal care work largely unexamined. Existing literature has given considerable attention to veterinary medicine (26–30) and demonstrates growing attention to shelter worker mental health (8, 31) contributing substantially to awareness of occupational distress in these populations. At the same time, other sectors within animal care have been dramatically overlooked. Animal control workers, despite being classified alongside police officers and firefighters in the highest-risk occupational group for workplace suicide (32) have been virtually absent from the empirical literature as a distinct population, with no published studies examining their psychological outcomes independent of the broader shelter worker category; existing research dedicated to this sector has focused on job realities rather than worker well-being specifically (33). Other underexamined populations include zoo and aquarium workers (34, 35), wildlife carers (36), laboratory animal care workers, pet care professionals such as groomers and trainers, and animal cremation and burial workers, none of whom have been studied with respect to moral injury. No study to date has systematically measured moral injury endorsement rates across the breadth of animal care sectors.

Animal care work shares the structural conditions associated with moral injury in military and healthcare populations: workers across sectors routinely face euthanasia decisions (2, 8), exposure to animal suffering and death (31, 36), resource constraints that force impossible choices in high-stakes situations (10), institutional limitations on ethical practice (37, 38), and challenging public interactions (38) — conditions that map directly onto the transgression-self, transgression-other, and betrayal pathways through which moral injury is theorized to occur (11).

The present study addresses these gaps by providing the first empirical examination of moral injury endorsement rates and phenomenology in a cross-sector sample of animal care workers. Using the MIOS (39) and the Patient Health Questionnaire-9 (PHQ-9), the author assessed morally injurious experience pathways, impact severity, functional impairment, and depression in 291 participants spanning rescue, shelter, animal control, veterinary medicine, volunteer/foster, and other animal care roles. The present study represents one of the first applications of these recently published caseness thresholds to any non-veteran population. Specifically, this study aimed to:

1. Estimate the frequency of morally injurious experiences across exposure pathways (witnessed, direct participation, and affected by others’ transgressions).
2. Characterize the severity and functional impact of moral injury in this population. Given the recent publication of norm-referenced caseness thresholds (17), the author further applied these criteria to distinguish moral distress from clinical moral injury and to examine subvariant presentations.
3. Examine sector differences in moral injury exposure rates, severity, and associated depression.
4. Investigate the differential relationships between moral injury dimensions (shame vs. trust violations) and psychological outcomes.

## Materials and methods

### Procedure

This cross-sectional survey study was reviewed for exempt research determination by Pearl IRB and determined to be Exempt according to 45 CFR 46.104(d)(2) on 06/11/2024 (IRB ID 2024-0249). Participants were recruited through convenience and snowball sampling via social media, animal care professional networks, and animal care organizations in the United States. Data were collected between June 2024 and July 2024. Participants provided informed consent electronically prior to beginning the survey. Inclusion criteria required active involvement in animal care work at the time of survey completion. No compensation was provided for participation, though participants had the option to register for a chance to win one of ten $25 Amazon gift cards.

The survey consisted of 106 items and included demographic items (age, gender, race/ethnicity, education, geographic location, years in animal care, weekly hours, species served), care role classification (14 options), an author-constructed 12-item exposure scale (analyzed in a separate publication; 40), the MIOS (comprised of one exposure item, 14 impact items and 8 functional impairment items), the PHQ-9, and additional validated measures analyzed in separate publications. Upon completion of the 106-item survey, participants were presented with an optional Part 2 survey consisting of an additional 99 items. Part 2 (n = 151) included additional validated measures and supplementary items, the results of which are reported in separate publications (38, 40). The present analyses draw exclusively from Part 1 data as a complete stand-alone investigation (N = 291).

### Participants

A total of 393 individuals accessed the survey; 390 provided informed consent. Five were excluded for not meeting the inclusion criterion of current involvement in animal care work. Of the remaining 385 respondents, 291 provided sufficient data to be included in the analytic sample. Complete data were available for all 291 participants on the PHQ-9. Ten participants did not complete the MIOS Impact items, yielding n = 281 for MIOS-based analyses, and five additional participants did not complete functional impairment items, yielding n = 286. The remaining 94 were excluded due to incomplete responses on one or more primary measures. Participants were 291 adults who self-identified as currently working or volunteering in animal care (**Supplementary Table 1**). The sample was predominantly female (64.9%, n = 189), White (81.4%, n = 237), with an age range from 18–80 and a mean age of 36.8 years (SD = 10.12). Participants reported their U.S. state or territory of residence, which was subsequently classified into four geographic regions using U.S. Census Bureau regional definitions. The South was most represented (42.8%, n = 124). The majority held a bachelor’s degree or higher (64.6%, n = 188). Staff members comprised 81.4% (n = 237) of the sample, with 10.3% (n=30) identifying as volunteer/foster only and 8.2% (n=24) in other animal care roles.

Participants indicated all animal care roles they currently held from a list of 14 options: full-time public shelter employee, part-time public shelter employee, full-time private rescue employee, part-time private rescue employee, animal control/investigation, shelter or rescue volunteer, foster, veterinarian, veterinary technician/assistant, other veterinary role, animal trainer/behaviorist, conservation-related, other animal care role with shelter interaction, and other animal care role without shelter interaction. These were consolidated into six primary sector categories for analysis: animal control, shelter (full- and part-time), veterinary medicine (veterinarian, veterinary technician, and other veterinary staff), rescue (full- and part-time), volunteer/foster, and other animal care roles (including training/behavior, conservation, and roles not fitting the preceding categories). For participants endorsing multiple roles (34.7%, n = 101), primary sector assignment was applied using a hierarchy informed by ecological systems theory (41), which conceptualizes human experience as shaped by nested environmental systems. Roles were prioritized by the extent to which they are structurally defined by multiple institutional systems including statutory legal mandates, governmental authority, professional licensure, and organizational formality, with the resulting hierarchy ranking animal control as the most institutionally embedded, followed by shelter, veterinary medicine, rescue, and volunteer/foster. This approach is consistent with the Job Demands-Resources model (42), which identifies organizational context as a moderator of the relationship between job demands and worker outcomes. Participants represented six primary sectors: rescue (29.9%, n = 87), shelter (22.7%, n= 66), animal control (18.2%, n = 53), veterinary medicine (10.7%, n = 31), volunteer/foster (10.3%, n = 30), and other animal care roles (8.2%, n = 24). Volunteers and fosters were included because they represent a substantial proportion of the animal care workforce, are exposed to many of the same occupational conditions and stressors as paid staff (36), and their inclusion enables examination of whether employment status moderates moral injury outcomes.

A majority had worked in animal care for 3 or more years (89.3%, n = 259), and the majority of participants reported substantial weekly engagement, with 62.4% (n = 181) working or volunteering 40 or more hours per week in animal care roles and 80.0% (n = 232) reporting 30 or more hours per week. Overall, 30.7% (n = 89) reported no hours in paid or volunteer work outside of animal care, suggesting that for many participants, animal care represented their primary or sole occupational role. Participants primarily worked with dogs and cats (81.1%, n = 236), though the sample also included workers serving wildlife (16.2%, n = 47), farm animals (16.2%, n = 47), exotic animals (10.3%, n = 30), marine animals (6.5%, n = 19), and research/laboratory animals (1.7%, n = 5), reflecting the diversity of the animal care workforce.

### Measures

#### Moral Injury Outcome Scale

The MIOS (39) was selected based on an extensive review of moral injury measurement approaches conducted during prior research on moral injury in military populations (16). Existing measures were evaluated on their capacity to separately assess exposure and syndrome impact, their applicability beyond military contexts, and their ability to capture dimensional variation in moral injury presentations. The MIOS was identified as the most suitable measure for cross-sector occupational research because: (a) it independently assesses both pMIE exposure pathways and moral injury impact as a syndrome, enabling prevalence estimation at both levels; (b) it was developed and validated across multinational civilian and military samples through the Moral Injury Outcome Scale Consortium (39, 43) establishing its applicability beyond military contexts; and (c) its two-subscale structure (Shame and Trust Violations) permits examination of differential mechanisms of harm. Following the 2024 data collection in this study, newly published norm-referenced caseness thresholds enabled improved and clinically meaningful severity classification not available for other measures (17).

The MIOS (39) is a self-report measure of morally injurious experiences and their psychological and functional impact. The exposure component consists of a single item presenting three pathway options from which participants could select all that applied: “I did something or failed to do something that went against my moral code or values” (hereafter DID), “I saw or witnessed something that went against my moral code or values” (hereafter SAW), and “I was directly affected by someone doing something or failing to do something that went against my moral code or values” (hereafter AFFECTED), along with a “None of the above” option. The Impact scale comprises 14 items rated on a 0 (strongly disagree) to 4 (strongly agree) scale, yielding a total score (range: 0–56) and two subscale scores: Shame (7 items; range: 0–28) and Trust Violations (7 items; range: 0–28). The Functional Impairment scale comprises 8 items rated on a 0 (not at all) to 6 (extremely) scale assessing impairment across life domains (work, relationships, spirituality, daily activities, education). In the present sample, internal consistency was excellent for the Impact Total (α = .906), Shame subscale (α = .883), Trust Violations subscale (α = .836), and Functional Impairment scale (α = .936).

In the standard MIOS administration protocol, participants who do not endorse any exposure pathway are not administered the impact or functional impairment items (39). In the present study, all participants were presented with the impact and functional impairment items for optional completion regardless of pathway endorsement. This departure from standard protocol was a deliberate design choice for four reasons: (a) as the first broad application of the MIOS to an animal care population, there was no prior basis for assuming the three pathway categories would comprehensively capture morally injurious experiences in this workforce; (b) presenting impact items to all participants enabled exploratory examination of potential measurement gaps in a population not previously studied; (c) the optional completion format preserved participants’ autonomy to skip items they deemed irrelevant while allowing those with clinical-level symptoms to report them regardless of pathway endorsement; and (d) given the theoretical possibility that moral distress may operate below full conscious awareness in chronically exposed populations, administering impact items regardless of pathway endorsement provided an empirical test of the author’s hypothesis that workers in morally saturated environments may internalize the effects of ongoing ethical violations without identifying discrete precipitating events.

#### Patient Health Questionnaire-9

The PHQ-9 (44) is a 9-item self-report measure of depressive symptom severity over the past two weeks, rated on a 0 (not at all) to 3 (nearly every day) scale. Total scores range from 0 to 27, with established clinical cutoffs of 5 (mild), 10 (moderate), 15 (moderately severe), and 20 (severe). The PHQ-9 demonstrated good internal consistency in the present sample (α = .887). The PHQ-9 was selected as the convergent validity comparator because depression has been consistently associated with moral injury across populations (13, 19) and represents the most commonly documented clinical correlate.

### Data analysis

#### Scoring and variable construction

MIOS Impact Total scores were computed as the sum of 14 impact items (range: 0–56). Shame and Trust Violations subscale scores were computed as the sum of their respective 7-item sets (range: 0–28 each). The MIOS Functional Impairment scale includes a not-applicable option for non-relevant life domains; functional impairment scores were calculated as mean scores across applicable items, consistent with B-IPF scoring procedures (45). PHQ-9 scores were computed as the sum of 9 items (range: 0–27). One participant did not respond to the MIOS exposure pathway item and was conservatively coded as not endorsing any pathway, as this participant completed all other survey items, suggesting engaged responding, and treating non-response as non-endorsement provides a conservative estimate. Sensitivity analyses excluding this participant did not alter any reported findings.

Recent caseness classification (17) was published subsequent to data collection and is applied here as the most current empirically supported framework for MIOS interpretation.^1^ The present analyses apply the norm reference T-score-derived thresholds for distinguishing among no clinically significant moral distress or injury, moral distress (subclinical, T = 60–64; raw scores 26–30), and moral injury (clinical syndrome, T ≥ 65; raw scores ≥ 31) (17). These thresholds were validated in a nationally representative veteran sample (N = 3,002) through concurrent comparison with established measures of PTSD, depression, functioning, and suicidality, demonstrating clear progressive differentiation across classification groups. Subvariant classification was determined by whether participants exceeded 1 SD above the sample mean on the Shame subscale, the Trust Violations subscale, both, or neither. Population-specific norms for animal care workers have not yet been established; the present study applies the veteran-derived raw score thresholds as the best available empirically supported framework for MIOS caseness classification.

#### Statistical analyses

All analyses were conducted in IBM SPSS Statistics (Version 29). Descriptive statistics were computed for all study variables, including means, standard deviations, skewness, and kurtosis. Moral injury exposure rates were calculated as the proportion of participants endorsing each MIOS exposure pathway and combinations thereof. Internal consistency was assessed using Cronbach’s alpha. One-way analyses of variance (ANOVA) with Tukey HSD *post-hoc* comparisons examined sector differences on MIOS scales and PHQ-9 scores, with eta-squared (η²) as the effect size measure. Levene’s test assessed homogeneity of variance; Welch’s robust test was applied where Levene’s test indicated unequal variances across groups. Normality was assessed within each sector group; skewness values ranged from -.657 to.658 and kurtosis values ranged from -1.618 to 1.311 across all sector × outcome variable combinations, all within acceptable ranges for parametric tests. Although some Shapiro-Wilk tests reached significance in larger subgroups, ANOVA is robust to moderate departures from normality with the group sizes in this study (n = 21–85). Bivariate Pearson correlations examined relationships among MIOS subscales, functional impairment, and depression. Chi-square analyses examined the association between each pMIE pathway and caseness classification independently; for each analysis, participants were classified as endorsing or not endorsing the specific pathway (e.g., DID endorsers vs. non-DID endorsers), creating independent groups within each test. Each chi-square test evaluated the null hypothesis that caseness classification was independent of pathway endorsement status, that is, the proportion of participants meeting each caseness threshold would not differ between those who endorsed a given pathway and those who did not. Because participants could endorse multiple pathways, these pathway-specific analyses are not independent of one another across tests. A separate chi-square examined the association between subvariant classification and caseness. Cramér’s V was used as the effect size measure for all chi-square analyses. Statistical significance was set at p <.05 for all tests.

## Results

### Descriptive statistics and scale properties

All MIOS and PHQ-9 distributions approximated normality, with skewness values ranging from.045 to.257 and kurtosis values ranging from –1.172 to –.016, supporting the use of parametric tests. Descriptive statistics for all study scales are presented in **Table 1**. The mean MIOS Impact Total score was 21.49 (SD = 10.45) on a 0–56 scale, representing 38.4% of the maximum score. The Trust Violations subscale mean (M = 11.88, SD = 5.51) exceeded the Shame subscale mean (M = 9.62, SD = 6.11) on the same 0–28 range, indicating that trust violation was the more commonly endorsed dimension of moral injury impact in this sample. The mean PHQ-9 score of 10.96 (SD = 6.27) exceeded the established clinical cutoff of 10 for moderate depression (44) indicating that the animal care workforce represented in this sample is experiencing clinically meaningful depressive symptomatology.

**Table 1:** Descriptive statistics and internal consistency for study scales (N = 291).

*Variable*	*N*	*M*	*SD*	*Range*	*Skewness*	*Kurtosis*	*α*
MIOS Total Score	281	21.49	10.45	0-56	.18	-.02	.906
MIOS Shame	281	9.62	6.11	0-28	.26	-.57	.883
MIOS Trust Violations	281	11.88	5.51	0-28	.06	-.15	.836
MIOS Functional Impairment	286	2.42	1.77	0-6	.13	-1.17	.936
PHQ-9 Depression	290	10.96	6.27	0-27	.05	-.56	.887

MIOS Functional Impairment scores reflect the item mean across 8 domains (0–6 scale). All other scores reflect scale totals.

Internal consistency was excellent across all MIOS scales (α range: .836–.906; see **Table 1**). These values are comparable to or exceed those reported by Litz et al. (39) in the multinational validation sample (α = .90 total) and by Litz et al. (17) in a nationally representative veteran sample (α = .89 total, .88 shame, .78 trust violation), supporting the reliability of the MIOS in a non-military occupational population.

### Aim 1: exposure frequency to morally injurious experiences

The vast majority of participants (83.5%, n = 243) endorsed at least one morally injurious experience pathway. Witnessing morally violative events was the most frequently endorsed pathway (63.9%, n = 186), followed by being affected by others’ transgressions (38.5%, n = 112) and direct participation (29.2%, n = 85). Of the total sample, 49.5% (n = 144) endorsed a single pathway, 19.9% (n = 58) endorsed two pathways, and 14.1% (n = 41) endorsed all three. The most common single-pathway endorsement was witnessing only (31.6%, n = 92). The most common multi-pathway combination was endorsement of all three pathways simultaneously (14.1%, n = 41), followed by witnessing combined with being affected by others’ transgressions (12.7%, n = 37). Exposure rates for all pathway combinations are presented in **Table 2**.

**Table 2:** Moral injury pathway endorsement and combinations (N = 291).

*Pathway*	*n*	*%*
Any pathway endorsed	243	83.5
Witnessed (SAW)	186	63.9
Affected by others’ transgressions (AFFECTED)	112	38.5
Direct participation (DID)	85	29.2
No pathway endorsed	48	16.5
Combination		
Single pathway		
SAW only	92	31.6
AFFECTED only	29	10.0
DID only	23	7.9
Single pathway subtotal	144	49.5
Two pathways		
SAW + AFFECTED	37	12.7
DID + SAW	16	5.5
DID + AFFECTED	5	1.7
Two pathway subtotal	58	19.9
Three pathways		
DID + SAW + AFFECTED	41	14.1

### Aim 2: MIOS severity, caseness, and subvariant classification

Among participants with valid MIOS scores (n = 281; 10 participants did not complete the impact items), the total score distribution was approximately normal (skewness = .18, kurtosis = −.02). Applying the 2025 (17) T-score-derived caseness thresholds to the full analytic sample, 61.6% (n = 173) showed no clinically significant moral distress or injury, 21.0% (n = 59) met criteria for moral distress (subclinical), and 17.4% (n = 49) met criteria for moral injury as a clinical syndrome. Among pMIE endorsers specifically (n = 243), 18.9% (n = 46) met criteria for moral injury and 21.4% (n = 52) for moral distress, with 59.7% (n = 145) falling below clinical thresholds.

Chi-square analyses examined the association between pMIE pathway endorsement and caseness classification among pMIE endorsers (n = 243). Endorsement of the agentic pathway (DID: “I did something or failed to do something”) was significantly associated with caseness, χ²(2) = 14.09, p <.001, Cramér’s V = .241. Among participants who endorsed the DID pathway (n = 85), 31.8% met criteria for moral injury, compared to 12.0% of those who did not endorse this pathway (n = 158). Neither the witnessing pathway (SAW), χ²(2) = 4.69, p = .096, Cramér’s V = .139, nor the directly affected pathway (AFFECTED), χ²(2) = 0.47, p = .792, Cramér’s V = .044, was significantly associated with caseness. The number of pathways endorsed (1–3) was not significantly associated with caseness, χ²(4) = 2.34, p = .673, Cramér’s V = .069, though the proportion meeting moral injury criteria increased descriptively from 16.0% among single-pathway endorsers (n = 144) to 22.4% for two pathways (n = 58) to 24.4% for three pathways (n = 41).

To examine the mechanism through which agentic pathway endorsement was associated with caseness, independent samples t-tests compared MIOS subscale scores between DID endorsers and non-endorsers from among the pMIE endorsers dataset (n = 243). DID endorsers scored significantly higher on both the Shame subscale (M = 12.22, SD = 5.82 vs. M = 9.15, SD = 5.77), t(241) = -3.95, p <.001, d = .53, and the Trust Violations subscale (M = 13.53, SD = 5.11 vs. M = 11.65, SD = 5.00), t(241) = -2.78, p = .006, d = .37. A chi-square analysis examining the association between DID endorsement and subvariant classification was significant, χ²(3) = 12.81, p = .005, Cramér’s V = .230. DID endorsers were three times more likely than non-endorsers to present with both subscales elevated (17.6% vs. 5.7%) and less likely to fall in the neither-elevated category (56.5% vs. 75.9%).

Among moral injury cases (n = 49), the majority presented with blended subvariant symptoms involving both shame-related and trust violation-related elevations (53.1%, n = 26), followed by shame-related only (26.5%, n = 13) and trust violation-related only (20.4%, n = 10). Among moral distress cases (n = 59), 64.4% (n = 38) did not meet the 1 SD threshold on either subscale, 23.7% (n = 14) showed shame-related elevations only, and 11.9% (n = 7) showed trust violation-related elevations only; no moral distress cases presented with both subvariants elevated.

The association between subvariant classification and caseness was highly significant, χ²(3) = 64.191, p <.001, Cramér’s V = .771, a large effect. All participants classified as having both shame and trust violation elevations met criteria for moral injury (n = 26, 100%), while all participants classified as having neither subscale elevated fell below clinical thresholds for moral injury, in the moral distress (subclinical) range (n = 38, 100%). Among single-subvariant presentations, shame-only cases were roughly evenly split between moral distress (51.9%, n = 14) and moral injury (48.1%, n = 13), while trust violation-only cases showed a slight clinical-leaning distribution (58.8% moral injury, n = 10; 41.2% moral distress, n = 7). The distribution of subvariant classifications across caseness categories are presented in **Table 3**.

**Table 3:** MIOS subvariant classification by caseness among participants meeting distress or injury thresholds (n = 108).

Subvariant	Moral distress n	(%)	Moral injury n	(%)
Neither elevated	38	(100.0)	0	(0.0)
Shame related only	14	(51.9)	13	(48.1)
Trust Violation Only	7	(41.2)	10	(58.8)
Both elevated	0	(0.0)	26	(100.0)
Total	59	(54.6)	49	(45.4)

χ²(3) = 64.191, p <.001, Cramér’s V = .771. Subvariant classification based on subscale scores ≥ 1 SD above the pMIE endorser mean (Shame ≥ 16; Trust Violations ≥ 17). Moral Distress = MIOS Impact Total 26–30; Moral Injury = MIOS Impact Total ≥ 31.

### Aim 3: sector differences in moral injury and depression

One-way ANOVAs revealed statistically significant sector differences on three of five outcome variables (**Table 4**). Sector accounted for the largest proportion of variance in functional impairment, F(5, 280) = 7.40, p <.001, η² = .117, a medium effect. Significant sector differences also emerged for MIOS Trust Violations, F(5, 275) = 2.96, p = .013, η² = .051 and PHQ-9 Depression, F(5, 284) = 3.31, p = .006, η² = .055. A fourth outcome variable, MIOS Impact Total, F(5, 275) = 2.30, p = .045, η² = .040 revealed an ambiguous effect that should be interpreted cautiously, as it showed statistically significant on one-way ANOVA but did not reach significance under Welch’s robust test (p = .060). Sector differences in the Shame subscale were not significant.

**Table 4:** Scale means by primary sector with ANOVA results.

Variable	Animal control M (SD) (n = 53)	Shelter M (SD) (n = 66)	Vet med M (SD) (n = 31)	Rescue M (SD) (n = 87)	Vol/foster M (SD) (n = 30)	Other M (SD) (n = 24)	F	p	η²
MIOS Total Impact	21.02 (10.26)	24.98 (10.99)	20.00 (11.34)	20.18 (9.98)	18.96 (8.42)	22.71 (10.58)	2.30	.045	.040
MIOS Shame	8.90 (6.02)	11.18 (6.47)	9.50 (6.56)	9.41 (5.80)	7.79 (4.80)	10.00 (6.82)	1.55	.173	.027
MIOS Trust Violations	12.12 (5.39)	13.80 (5.44)	10.50 (5.86)	10.76 (5.24)	11.18 (5.32)	12.71 (5.59)	2.96	.013	.051
MIOS Functional Impairment	2.50 (1.71)	2.47 (1.43)	2.08 (1.57)	2.99 (1.98)	0.84 (1.09)	2.31 (1.79)	7.40	<.001	.117
PHQ-9 Depression	10.13 (6.53)	12.98 (6.47)	9.03 (6.89)	11.67 (5.35)	9.38 (6.89)	9.04 (5.08)	3.31	.006	.055

Sector n’s reflect primary sector classification. Analytic n’s vary slightly across measures due to missing data (total analytic n: MIOS scales = 281, Functional Impairment = 286, PHQ-9 = 290). Functional Impairment reflects the item mean (0–6 scale). Significant Tukey HSD comparisons: VF < AC, SH, RE, and OT on Functional Impairment (all p <.05); SH > RE on Trust Violations (p = .010); SH > VM on Depression (p = .040). Levene’s test was significant for Functional Impairment (p <.001); Welch’s F(5, 96.09) = 12.30, p <.001 confirmed the result. Impact Total reached significance under standard ANOVA but not under Welch’s test (p = .060).

Tukey HSD *post-hoc* comparisons identified three specific pairwise differences. First, volunteer/foster workers reported significantly lower functional impairment than animal control, shelter, rescue, and other sector workers (all p <.05), emerging as a distinct homogeneous subset. Second, shelter workers reported significantly higher trust violations than rescue workers (p = .010). Third, shelter workers reported significantly higher depression than veterinary medicine workers (p = .040). Across all measures, shelter workers reported the highest mean scores while volunteer/foster workers reported the lowest.

### Aim 4: correlations and convergent validity

Bivariate correlations among study variables are presented in **Table 5**. The MIOS Shame and Trust Violations subscales were moderately to strongly correlated (r = .617, p <.001), supporting the two-subscale structure while confirming they capture related but distinct constructs.

**Table 5:** Bivariate correlations among study variables.

Variable	M	SD	1	2	3	4	5
1. MIOS Impact Total	21.49	10.45	:				
2. MIOS Shame	9.62	6.11	.910**	:			
3. MIOS Trust violations	11.88	5.51	.888**	.617**	:		
4. MIOS Functional Impairment	2.42	1.77	.224**	.343**	.045	:	
5. PHQ-9 depression	10.96	6.27	.621**	.646**	.462**	.424**	:

N = 281–290 due to pairwise deletion. MIOS Functional Impairment reflects the item mean (0–6 scale). **p <.01 (all significant correlations were p <.001 except Trust Violations × Functional Impairment, which was nonsignificant.).

The MIOS Impact Total demonstrated strong convergent validity with the PHQ-9 (r = .621, p <.001), as did both subscales: Shame (r = .646, p <.001) and Trust Violations (r = .462, p <.001). These correlations indicate meaningful association between moral injury and depression without suggesting construct redundancy (r <.80). A divergent pattern emerged in the relationship between MIOS subscales and functional impairment. Shame was significantly correlated with functional impairment (r = .343, p <.001), whereas Trust Violations was not (r = .045, p = .456).

## Discussion

This study provides the first empirical estimate of moral injury outcomes, using a validated syndrome-specific measure (MIOS), in a large cross-sector sample of animal care workers. The following discussion addresses each study aim in turn, beginning with pathway prevalence estimates and phenomenology (Aim 1), followed by caseness and subvariant classification (Aim 2), sector differences (Aim 3), and differential relationships between moral injury dimensions and outcomes (Aim 4), before considering implications across aims.

The finding that 83.5% of participants endorsed at least one morally injurious experience pathway suggests not only that moral injury may be a common occupational experience in this workforce, but that it may represent a significant and previously unexamined dimension of occupational harm in this population distinct from frameworks that have historically dominated the animal care literature. Notably, this endorsement rate is higher than rates reported in nationally representative military samples (17), though direct comparison is limited by differences in sampling methodology.

Application of the 2025 (17) caseness thresholds, the first known use of these criteria outside a veteran population, revealed that 17.4% of the overall sample (18.9% of pMIE endorsers) met criteria for moral injury as a clinical syndrome and an additional 21.0% of the sample (21.4% of pMIE endorsers) met criteria for moral distress. These rates are clinically meaningful: nearly one in five animal care workers in this sample carried a clinical-level moral wound, and two in five showed at least subclinical moral distress. Direct comparison with the Litz et al. (17) veteran prevalence data (13.1% moral injury among pMIE endorsers) is complicated by differences in sampling (nationally representative veterans vs. self-selected animal care workers), but the application of identical caseness criteria to both populations enables a methodologically grounded point of reference that has not previously been available for this workforce. Notably, the moral injury caseness rate among pMIE endorsers in the present sample (18.9%) exceeded the corresponding rate in the veteran sample (13.1%), suggesting that moral injury may be at least as prevalent among animal care workers as among veterans, though this comparison should be interpreted with caution given differences in sampling methodology.

It should be noted that the MIOS AFFECTED item [“I was directly affected by someone doing something or failing to do something that went against my moral code or values (e.g., being betrayed by someone you trusted)”] may reflect both exposure and response, unlike the more clearly event-based DID and SAW items. This measurement consideration should be weighed when interpreting the 83.5% overall exposure rate.

### Pathways to moral injury in animal care

The dominance of the witnessing pathway (63.9%) over direct participation (29.2%) is theoretically significant, and parallels the military literature, where transgression-self events are consistently least endorsed (46–48). Witnessing was the most prevalent component across virtually all pathway configurations: as the most endorsed single pathway (31.6%), the leading element in two-pathway combinations (witnessing plus being affected, 12.7%; witnessing plus direct participation, 5.5%), and a constituent of all three-pathway endorsements (14.1%). This pervasiveness suggests that witnessing morally violative events is a prevalent feature of the occupational landscape, indicating that animal care workers may operate in morally saturated work environments where they are routinely exposed to suffering, death, and ethically compromising decisions made by others (2, 30, 31, 36, 37), regardless of their own level of direct involvement. While it is possible that the low endorsement of direct participation may reflect occupational reality, it should be acknowledged that there is also stigma and self-protective avoidance associated with identifying oneself as an agent of harm, even when systemic constraints compel such actions (16, 49). Several psychological mechanisms may contribute to underreporting of agentic moral injury. Alexithymia — difficulty identifying and describing one’s own emotional states — has been documented as a persistent factor in trauma-exposed populations (50), and dissociative responses to morally distressing experiences, social sanctions and identity threat, self-protective denial, and the absence of psychological safety (51) in workplace environments may further limit the accuracy of self-report measures that require individuals to recognize and label their internal experiences. In animal care contexts, emotional suppression may be reinforced by workplace norms that discourage expressions of distress (37), potentially leading workers to underreport agentic moral injury even when they have engaged in morally violative acts. It should be noted that these mechanisms have been primarily studied in the context of fear-based trauma and PTSD rather than moral injury specifically, and whether they operate similarly in the context of moral and ethical violations remains an open question. Their consideration here reflects the theoretical plausibility of their relevance to morally injurious contexts, which warrants empirical investigation. This possibility is consistent with the methodological rationale for administering MIOS impact items to all participants regardless of pathway endorsement, as workers unable to identify or articulate their exposure pathway may nonetheless carry measurable moral injury impact.

Beyond the question of underreporting, the pathway data also reveal that a substantial proportion of workers experience moral injury exposure across multiple dimensions simultaneously. Endorsement of all three pathways simultaneously (14.1%) was the most common multi-pathway combination, suggesting that a substantial subgroup of animal care workers experiences comprehensive moral injury exposure, simultaneously doing, seeing, and being affected by morally violative acts. This cumulative exposure pattern may reflect the structural reality of animal care settings, where workers are embedded in systems that require them to participate in morally conflicting acts, witness harm they cannot prevent, and experience the consequences of others’ decisions, often within the same organizational context and sometimes within the same workday. This pattern resonates with what has been described as the caring-killing paradox in shelter contexts (37) and may additionally represent a form of institutional betrayal, where workers observe violations of the very values their organizations publicly espouse.

### The agentic pathway and clinical severity

The finding that the agentic pathway (DID) was the only exposure pathway significantly associated with caseness carries theoretical weight. Workers who identified themselves as having done or failed to do something morally violative were nearly three times more likely to meet criteria for clinical moral injury than those who did not endorse this pathway (31.8% vs. 12.0%), while neither witnessing nor being affected by others’ transgressions was significantly associated with caseness. This parallels Litz et al.’s (17) finding of elevated odds of moral injury among agentic pMIE endorsers in veterans and aligns with the broader literature suggesting that perpetration-based or complicity-based moral injury carries a particularly potent psychological burden (11).

Exploratory analyses examining the mechanism underlying this association revealed that agentic pathway endorsement was associated with significantly elevated scores on both the Shame and Trust Violations subscales, not shame alone. DID endorsers were three times more likely than non-DID endorsers to present with the blended subvariant, a co-elevation of both shame and trust violations (17.6% vs. 5.7%, p = .005). This suggests that the agentic pathway may be uniquely potent because it simultaneously activates both self-directed moral pain and other-directed relational rupture. Workers who perceive themselves as having done or failed to do something morally violative may internalize blame for the act while simultaneously recognizing the systemic conditions that compelled it, producing the dual-dimension injury pattern that characterizes the majority of clinical cases in this sample. This finding refines the theoretical understanding of why agentic moral injury carries greater clinical severity: it may not be simply that perpetration produces more shame, but that the experience of constrained moral agency generates a cascading injury across both the internal and relational dimensions of moral functioning.

In animal care contexts, the agentic pathway encompasses not only direct acts such as performing euthanasia but also perceived failures to act such as not intervening when witnessing poor animal handling, not advocating against policies experienced as harmful, or not leaving an organization whose practices conflict with one’s values. The significance of perceived moral agency in determining clinical outcomes suggests that the mechanism through which pMIEs produce clinical-level harm may depend more on the individual’s perceived role in the event than on the event type itself. Interventions targeting self-forgiveness, moral repair, and the reappraisal of constrained agency may be particularly important for animal care workers at highest risk.

### Caseness, severity, and subvariant classification

The severity and subvariant findings extend the Litz et al. (17) caseness framework to a non-military occupational population and reveal patterns with both psychometric and clinical implications. The strong association between subvariant classification and caseness (Cramér’s V = .771) requires careful interpretation, as the two classifications are not statistically independent. Caseness is determined by total MIOS score thresholds, and subvariant classification is based on subscale elevations that contribute to that total; co-elevation of both subscales therefore virtually guarantees scores in the clinical range, while absence of elevation on either subscale constrains scores below caseness thresholds. The perfect mapping observed for these categories — 100% of both-elevated cases meeting moral injury criteria and 100% of neither-elevated cases falling in the subclinical range — reflects this mathematical interdependence rather than independent empirical validation.

However, this structural relationship carries clinical meaning. Individuals with elevations on both shame and trust violation have lost both their internal psychological foundation in terms of self-regard, moral identity, and the belief that one is a good person and their external relational foundation pertaining to trust in others, institutions, and leadership (17, 52, 53). The convergence of these two forms of injury may represent a qualitatively different and more entrenched clinical presentation than either dimension alone, a possibility that warrants investigation through longitudinal and treatment-outcome research.

The single-subvariant presentations, which are not constrained by this mathematical relationship, provide genuinely informative data about the differential clinical weight of each dimension. Trust violation-only cases leaned toward clinical-level moral injury (58.8% moral injury, 41.2% moral distress), while shame-only cases were nearly evenly split slightly favoring moral distress (48.1% moral injury, 51.9% moral distress). At the same time, the correlational analyses revealed an opposing pattern for functional impairment: shame was significantly associated with functional impairment (r = .343, p <.001), whereas trust violations was not (r = .045, p = .456). Rather than contradictory, these divergent patterns suggest that shame and trust violation operate through distinct mechanisms that produce different forms of harm.

### Shame, trust, and differential mechanisms of harm

The clinical leaning of trust violation-only presentations may reflect the fundamentally social-relational nature of moral injury as a syndrome. Moral injury can be conceptualized as a syndrome embedded within social-relational contexts and activated through the interplay of social sanctions and internalized self-sanctions, a theoretical framework grounded in social cognitive theory, identity theory, and moral psychology that has been applied to moral injury conceptualization (16, 53, 54). Trust violations, which are inherently interpersonal and embedded within human-made systems of authority, obligation, and care, may therefore represent a more direct pathway to the clinical constellation of moral injury impact.

The association between shame and functional impairment, by contrast, may reflect the distinct behavioral consequences of shame as a moral emotion. Guilt and shame, though often conflated, produce divergent action tendencies with distinct implications for functioning (52). Guilt, as an adaptive moral emotion, activates prosocial and reparative behaviors (confession, behavioral change, attempts at repair) that preserve social bonds and protect moral identity. Shame, however, represents a maladaptive consolidation in which the negative evaluation shifts from the behavior (“I did a bad thing”) to the self (“I am bad”), activating withdrawal, concealment, and avoidance rather than approach and repair (52). These withdrawal-oriented responses directly compromise daily functioning by disrupting engagement with work, relationships, and self-care, which may explain why shame was uniquely associated with functional impairment in the present sample.

Notably, this functional disruption occurred across the severity spectrum, including at subclinical levels, suggesting that shame-driven withdrawal may begin eroding daily functioning before moral injury fully consolidates as a clinical syndrome. Among subclinical moral distress cases showing any dimensional loading, shame-related presentations (23.7%) were more common than trust violation-related presentations (11.9%), suggesting that workers and organizations should be attentive to shame-related signs of moral distress (withdrawal, concealment, and disengagement) as potential early indicators of functional decline even before clinical thresholds are reached. The MIOS impact items that comprise the shame subscale capture this internalized dimension directly (e.g., “I feel like I don’t deserve a good life,” “I am not the good person I thought I was”), reflecting the global, stable self-attributions characteristic of shame rather than the behavior-specific appraisals characteristic of adaptive guilt. The stronger association of shame with depression (r = .646 vs. r = .462 for trust violations) further aligns with cognitive models in which shame-based cognitions, particularly global, stable attributions about one’s moral character, drive depressive symptomatology (52, 55).

This distinction carries practical implications for intervention at both the individual and organizational levels. Addressing shame may be more urgent for preserving daily functioning, while addressing trust violation may be more critical for preventing clinical escalation. If organizational strategies target only trust-rebuilding — improving leadership transparency, communication, and institutional accountability — without also addressing internalized shame, they may miss the workers most at risk for functional decline. Conversely, clinical interventions targeting self-forgiveness, self-compassion, and cognitive processing of moral self-appraisals, while necessary, are insufficient if deployed to the exclusion of organizational accountability and reform. Organizations that address individual shame without reforming the conditions that produce moral injury remain structurally susceptible to continued moral injury and, at minimum, moral distress, even among workers who have undergone individual treatment.

### Blended subvariant presentation in clinical moral injury

The predominance of blended subvariant presentations among moral injury cases (53.1%) further distinguishes the phenomenology of moral injury in animal care from the pattern observed in military populations, where Litz et al. (17) reported a predominantly shame-related presentation (47.8%) with fewer blended cases (33.3%). Animal care workers who reach clinical thresholds are more often carrying both the internalized weight of shame and the relational rupture of trust violation simultaneously. This divergence may reflect fundamental differences in relational context of morally injurious events across these populations. In military settings, a substantial proportion of morally injurious acts occur in relation to combat adversaries (11, 46, 48), which may partially buffer the trust violation dimension even when shame is profound. While betrayal by leadership and fellow service members is well-documented in military moral injury literature (11, 13, 17), the military context also provides a framework in which some morally injurious acts can be attributed to external threat or enemy action. Animal care workers have no such framework available. Trust violations in this population originate almost exclusively from within the worker’s own professional systems — from organizations that espouse the same values workers hold and from the communities they live in and the public they serve — thereby activating identity threats unique to violations of trust within one’s own moral community (16, see also 53, 54, 56). This relational context converges with the structural demands of the work itself: animal care workers frequently engage in actions that conflict with their values (e.g., euthanasia decisions, triage under resource constraints; 2, 8) while simultaneously operating within organizational cultures characterized by hierarchical dysfunction, inadequate institutional support, and interpersonal conflict (38). The simultaneous presence of shame-producing actions and trust-violating conditions within the same occupational context may explain why blended presentations predominate among animal care workers who reach clinical thresholds.

### Sector differences and the role of autonomy

Shelter workers consistently reported the highest levels of moral injury impact, trust violations, and depression across sectors, while volunteer and foster workers reported significantly lower functional impairment than animal control, shelter, rescue, and other sector workers, emerging as a distinct homogeneous subset. This pattern is notable given prior findings that volunteers experience organizational stressors at rates comparable to paid staff (38). If stressor exposure is similar but functional outcomes diverge, the protective factor may not be reduced exposure but rather preserved autonomy, moral agency, reduced institutional embeddedness, or other yet unidentified variables.

Volunteers retain the ability to modulate their involvement, stepping back, reducing hours, or disengaging when the moral weight or institutional misalignment intensifies, without the financial consequences that constrain paid workers. This capacity to titrate exposure, before subclinical symptoms consolidate, may buffer against the functional impairment that accumulates among those who cannot exit morally injurious conditions as easily. Shelter settings characterized by open-intake policies constrain professional autonomy in ways that limited-intake organizations, which can manage caseloads selectively, do not.

Shelter workers reported the highest scores across nearly all measures, with significantly higher trust violations than rescue workers and significantly higher depression than veterinary medicine workers. This pattern suggests that the institutional structures of shelter settings may generate a distinct form of institutional betrayal, in which the combination of constrained autonomy, high-stakes moral decisions, and inadequate organizational support makes trust violation a predictable occupational outcome rather than an aberration. Animal control workers, while not reaching statistical significance in pairwise comparisons, trended in a similar direction to shelter workers and notably reported functional impairment (M = 2.50) virtually identical to shelter workers (M = 2.47) despite lower moral injury impact and shame scores. This dissociation between functional impairment and moral injury severity in this population may operate through mechanisms not fully captured by the MIOS; the possibility that enforcement-oriented cultures influence underreporting of moral injury, or that enforcement framing of animal control work provides a degree of moral agency protection—as shame exposures may be counterbalanced by one’s ability to take specific action against perceived wrongdoing and effect outcomes—warrants further investigation.

### pMIE non-endorsers and the limits of pathway categories

The present study departed from standard MIOS administration protocol, which directs non-endorsers past the impact and functional impairment items (39), by presenting these items to all participants regardless of pathway endorsement. This design choice proved empirically informative. Of the 48 participants who did not endorse any pMIE pathway, 38 completed the impact items and 43 completed functional impairment items. Among these non-endorsers, 7 met thresholds for moral distress and 3 met caseness criteria for moral injury as a clinical syndrome. The mean impact score among non-endorsers who completed these items was 14.87 (range 0–41), indicating that non-endorsers were not uniformly scoring at floor levels but were endorsing moral injury impact items at mild levels despite denying pathway exposure.

The three non-endorsers who met clinical caseness thresholds (Impact Total scores of 34, 36, and 41) are particularly noteworthy because all three had actively selected ‘None of the above’ on the pathway item; they did not skip the question or leave it blank, but explicitly denied exposure to any of the three pMIE categories while nonetheless reporting clinical-level impact. This pattern is unlikely to reflect random responding, as scores of this magnitude (34, 36, and 41 on a 0–56 scale) require consistent endorsement across multiple impact items. Several explanations warrant consideration. These individuals may be experiencing moral injury impact but do not conceptualize their experiences as fitting the pathway categories offered. Alternatively, they may be minimizing or denying exposure while carrying its impact, a pattern consistent with the avoidance and concealment that characterize shame-based moral injury responses. It is also possible that in morally saturated environments such as animal care settings, ethically distressing conditions become normalized within daily institutional rhythms to the degree that workers internalize their effects without identifying a discrete precipitating event, consistent with the author’s hypothesis that moral distress may operate below full conscious awareness in chronically exposed populations.

It is also possible that these individuals are experiencing shame and trust violation from sources unrelated to morally injurious events — such as general occupational stress, interpersonal conflict, or preexisting psychological vulnerability — and that their MIOS scores reflect a broader distress response rather than moral injury specifically. This possibility underscores the importance of the MIOS pathway-then-impact administration sequence and suggests that interpreting impact scores in the absence of pathway endorsement requires clinical judgment.

While preliminary and requiring replication with larger non-endorser samples, this observation raises the possibility that the three MIOS pathway categories may not capture all experiential routes through which moral injury develops in occupational populations where morally injurious conditions are ambient and ongoing rather than linked to identifiable critical incidents. This finding supports continued refinement of moral injury exposure assessment for these contexts and suggests that administration of impact items regardless of pathway endorsement may yield clinically meaningful information that would otherwise be lost.

### Moral injury among adjacent occupational health constructs

The sample mean PHQ-9 score of 10.96 exceeded the clinical threshold for moderate depression, indicating that the animal care workforce represented in this sample is in clinically significant distress. While the cross-sectional design precludes causal interpretation, the strong correlation between moral injury severity and depression (r = .621) suggests these conditions share substantial variance. Importantly, the shared variance (~39%) leaves approximately 61% of the variance in moral injury unaccounted for by depression alone, supporting the distinctiveness of the moral injury construct and reinforcing the argument that depression screening alone is insufficient for capturing the moral dimensions of occupational harm in this population.

The differential associations between MIOS subscales and depression further illuminate this distinction. Shame was more strongly associated with depression (r = .646) than trust violations (r = .462), a pattern consistent with cognitive models in which global, stable self-attributions about one’s moral character drive depressive symptomatology (52, 55). That shame was also uniquely associated with functional impairment (r = .343) while trust violations were not (r = .045) suggests that the mechanisms linking moral injury to daily functioning operate through different pathways than those linking moral injury to depressive severity — a specificity that would not be captured by general mental health screening.

While burnout, compassion fatigue, and secondary traumatic stress were not analyzed in the present study — these constructs are examined in a separate analysis from this dataset (40)—the patterns observed here are consistent with growing recognition that moral injury operates through mechanisms distinct from those captured by existing occupational health frameworks (13, 19, 21). The shame dimension of moral injury, which captures internalized moral self-evaluation and drives functional withdrawal, operates through moral-evaluative mechanisms that are not addressed by existing occupational health frameworks focused on workload, emotional exhaustion, or vicarious exposure. Similarly, trust violation reflects relational and institutional rupture rather than vicarious trauma exposure or resource depletion. These distinctions carry practical implications: interventions designed to address workload reduction or emotional processing may not reach the moral wound at the core of shame-based functional decline, nor repair the institutional trust that has been violated.

## Limitations

Several limitations warrant consideration. First, the cross-sectional design precludes causal inferences; prospective studies are needed to determine whether morally injurious experiences precede the development of moral injury symptoms and depressive outcomes, or whether preexisting psychological vulnerability increases susceptibility to moral injury. Second, the self-selected, convenience sample may overrepresent those most distressed by their work, potentially inflating exposure frequency estimates. Third, the sample was predominantly White (81.4%) and female (64.9%), limiting generalizability; racial and ethnic minority animal care workers may experience distinct forms of moral injury intersecting with systemic racism and discrimination within animal care institutions. Fourth, the online survey format and recruitment through professional networks and social media may underrepresent workers in under-resourced settings who have less institutional connectivity, limited engagement with professional organizations, and fewer opportunities to encounter research participation invitations; these populations may be at greatest risk for moral injury due to the very resource constraints that limit their visibility in research. Fifth, this study did not include a comparison group, precluding assessment of whether moral injury rates in this population exceed those in the general working population.

Sixth, the Litz et al. (17) caseness thresholds were derived from T-score distributions in a nationally representative veteran sample, and population-specific norms for animal care workers do not yet exist. While the application of standardized cutoffs across populations is consistent with established practice for clinical measures (e.g., PHQ-9), the absence of animal care-specific normative data should temper definitive conclusions about caseness rates until population-specific validation is available. Seventh, only PHQ-9 depression outcomes were examined in relation to the MIOS in this analysis; subsequent analyses examining the relationship between moral injury and compassion fatigue, burnout, and secondary traumatic stress are reported elsewhere (40). Additionally, PTSD was not assessed in the present study, as the focus was on moral injury as distinct from fear-based posttraumatic responses.

## Future directions

Future research should pursue longitudinal designs tracking the trajectory of moral injury across career stages, discriminant validity analyses examining the MIOS against established burnout and secondary traumatic stress measures, and organizational predictor models identifying institutional factors that increase or buffer moral injury risk. Qualitative investigation of the lived experience of moral injury in animal care, particularly exploration of the blended shame-and-trust-violation presentation that characterized the majority of clinical cases, would provide phenomenological depth to the quantitative patterns identified here. The finding that agentic pathway endorsement was associated with disproportionate representation in the blended subvariant category, suggesting that the DID pathway may predict caseness through simultaneous activation of both shame and trust violation dimensions rather than through shame alone, warrants replication in larger samples and prospective designs that can establish temporal sequencing between pathway exposure, subscale elevation, and caseness onset. The development of targeted interventions addressing shame-based moral injury, particularly in shelter settings where structural constraints limit individual coping capacity, represents an urgent applied priority. Examination of protective factors, including professional autonomy, organizational voice, and peer support structures, may identify modifiable organizational conditions that reduce moral injury risk without placing the burden of adaptation solely on workers.

The present study’s cross-sector design enabled comparison across rescue, shelter, animal control, veterinary medicine, and volunteer/foster workers, but several animal care populations remain underrepresented in the moral injury literature. Laboratory animal care workers, slaughterhouse and meatpacking workers, wildlife and conservation professionals, pet care professionals, and animal cremation and burial workers may experience morally injurious conditions that are qualitatively distinct from those in companion animal care, yet no study has examined moral injury in these populations. Animal control workers, despite their inclusion in the present sample, remain one of the least studied populations in the occupational mental health literature and warrant continued focused investigation.

Future investigations should also consider whether moral injury exposure language developed in military contexts adequately captures the range of morally injurious experiences in animal care. Terms like ‘perpetration’ and ‘transgression’ may resonate differently across animal care contexts depending on the worker’s perceived moral agency, the availability of moral justification for the act, and the degree to which the act is experienced as compelled by systemic constraints versus chosen. The present finding that DID endorsers experienced the highest caseness rates suggests that agentic moral injury language does capture meaningful variance in this population, but the specific moral frameworks through which animal care workers interpret their agency — including contexts where acts causing harm are simultaneously understood as acts of care — warrant further examination. Additionally, ongoing discussion in the moral injury literature about the optimal categorization of pMIE exposure may have implications for animal care populations specifically. In past discourse, arguments have been made for a three-factor model (transgression-self, transgression-other, betrayal (15), a two-factor model collapsing transgression-other and betrayal (47) or collapsing both transgression types (57), or a commission/omission distinction (23), all of which has implications for how exposure is conceptualized and measured. The present finding that some pMIE non-endorsers nonetheless met caseness thresholds suggests that further attention to exposure categorization and measurement is warranted, particularly for occupational populations in morally saturated environments in which moral injury may arise from chronic systemic conditions rather than discrete events. Relatedly, future research should examine whether the AFFECTED pathway conflates exposure and outcome or functions equivalently to the more event-specific DID and SAW pathways in predicting clinical outcomes.

## Conclusion

Moral injury is a prevalent and consequential occupational experience among animal care workers, with exposures occurring for more than four in five workers in this sample and reaching clinical severity in nearly one in five and subclinical levels in an additional one in five. The findings suggest that moral injury represents a dimension of animal care worker harm that has been largely unnamed, unmeasured, and unaddressed in existing occupational health research. The disproportionate burden borne by shelter workers, the critical role of shame in functional decline, the distinct contribution of trust violation to clinical severity, and the construct validity of the MIOS caseness framework in this population point toward specific organizational and clinical intervention targets. Moral injury in animal care is not simply an individual clinical problem; it is a structural feature of systems that ask workers to enact values they do not hold, witness suffering they cannot prevent, and absorb the moral costs of institutional decisions made above them. Naming it is the first step toward repairing it.

## Data Availability

The dataset presented in this article is not readily available because the data are part of an independently funded and ongoing research program with additional analyses and publications in progress. Requests to access the dataset should be directed to info@fortifyucoaching.com and will be considered on a case-by-case basis subject to a data sharing agreement.

## Footnotes

1 Preliminary severity findings from this dataset were first presented using the 2022 MIOS scoring guidelines at the 2024 International Association of Veterinary Social Work Summit (McNally, J, conference presentation, 2024).
