# Supplementary material for: Moral injury in animal care workers: prevalence, pathways, and phenomenology in a cross-sector sample
Source: Front Psychiatry. 2026 Jun 17;17:1820899. doi: 10.3389/fpsyt.2026.1820899 (PMC13320660; doi:10.3389/fpsyt.2026.1820899)
Supplement: Supplementary file 1 [file Table1.docx]

**Table 1**

*Demographic Characteristic of Animal Care Workers (N=291)*

| ***Demographic Characteristic*** | ***n* or M** | ***% or SD*** |
| --- | --- | --- |
| Age (years) | 36.82 | 10.12 |
| Age group |  |  |
| 18-29 | 60 | 21.1 |
| 30-39 | 138 | 48.4 |
| 40-49 | 59 | 20.7 |
| 50-59 | 16 | 5.6 |
| 60+ | 12 | 4.2 |
| Gender Identity |  |  |
| Female | 189 | 64.9 |
| Male | 86 | 29.6 |
| Non-binary/genderqueer | 10 | 3.4 |
| Agender | 2 | 0.7 |
| Prefer not to answer | 1 | 0.3 |
| Race/Ethnicity |  |  |
| American Indian/Alaska Native | 12 | 4.1 |
| Asian/Pacific Islander | 6 | 2.1 |
| Black/African American | 17 | 5.8 |
| Hispanic/Latino | 9 | 3.1 |
| White/Caucasian | 237 | 81.4 |
| Prefer not to answer | 3 | 1 |
| Education |  |  |
| High school/GED or less | 19 | 6.5 |
| Some college/associate degree | 83 | 28.5 |
| Bachelor’s degree | 88 | 30.2 |
| Master’s Degree | 75 | 25.8 |
| Doctoral or professional degree | 25 | 8.6 |
| Prefer not to answer | 1 | 0.3 |
| U.S. Geographic Region |  |  |
| Northeast | 37 | 12.8 |
| Midwest | 52 | 17.9 |
| South | 124 | 42.8 |
| West/territories | 77 | 26.6 |
| Years in Animal Care |  |  |
| Over 20 | 26 | 9 |
| 10-19 | 75 | 25.9 |
| 5-9 | 98 | 33.8 |
| 3-4 | 60 | 20.7 |
| 1-2 | 21 | 7.2 |
| Less than a year | 10 | 3.4 |
| Animal Care Roles (primary sector) |  |  |
| Animal Control | 53 | 18.2 |
| Shelter | 66 | 22.7 |
| Vet Med | 31 | 10.7 |
| Rescue | 87 | 29.9 |
| Volunteer/Foster | 30 | 10.3 |
| Other | 24 | 8.2 |
